# Simulation of stroke gait impairment correction using cable-driven lower limb rehabilitation exoskeleton (C-LREX)

**DOI:** 10.1017/wtc.2025.10013

**Published:** 2025-08-08

**Authors:** Rajan Prasad, Marwan El-Rich, Mohammad I. Awad, Kinda Khalaf

**Affiliations:** 1 Department of Mechanical Engineering, Khalifa University, Abu Dhabi, United Arab Emirates; 2 Healthcare Engineering and Innovation Center (HEIC), Khalifa University, Abu Dhabi, UAE; 3 Electrical, Computer and Biomedical Engineering Department, College of Engineering, Abu Dhabi University, Abu Dhabi, UAE; 4 Department of Biomedical Engineering, Khalifa University, Abu Dhabi, UAE

**Keywords:** exoskeleton, lower limb rehabilitation, cable-driven exoskeleton, stroke rehabilitation, cable routing, trajectory tracking

## Abstract

Cable-driven exoskeletons have recently shown great promise in the rehabilitation of stroke survivors. Numerical modeling/simulation provides a cost- and time-effective approach to fine-tuning design parameters of the exoskeletons, hence reducing the need for expensive and time-consuming experimental trials. This study investigated using a cable-driven lower limb rehabilitation exoskeleton (C-LREX) to correct stroke-impaired gait and track reference healthy trajectories. The impact of different levels of impairment and subject anthropometry variation on the model’s performance was studied. The C-LREX model was successful in assisting the impaired limb to track the reference trajectory in all impaired gait patterns, except for higher impairment levels (>20° range of motion deviation at the hip joint). Subject anthropometry variation did not affect trajectory tracking when the cable routing was scaled to fit the user’s anthropometry. This study confirmed that the C-LREX model could simulate various impaired lower limb gait patterns in the sagittal plane and determine the cable tension requirements needed to correct the impairment. Future work includes expanding the framework to incorporate frontal plane motion and to validate C-LREX performance in assisting biplanar impaired motion.

## 1. Introduction

Stroke is considered the leading cause of disability and the second leading cause of death worldwide (Johnson et al., 2016) by the World Health Organization. The Global Stroke Factsheet released in 2022 reveals that the lifetime risk of developing a stroke has increased by 50% over the last 17 years, where currently, it is estimated that one in four people will have a stroke in their lifetime (Feigin et al., 2022). Hemiparesis, or the partial paralysis of one side of the body, is a prevalent challenge experienced by stroke survivors. The resulting impairment is reflected in changes to the normal gait cycle, including a reduction in the range of motion (ROM) of joints, such as the ankle, knee, and hip, as well as the development of muscle spasticity and abnormal patterns of muscle activation (Balaban and Tok, 2014; Sheffler and Chae, 2015).

Robotic rehabilitation using exoskeletons has recently emerged as a promising technology, as it offers consistent, repetitive, and intensive training compared to manual therapy. In the literature, various exoskeletons, including link-driven (Ullas and Rajendrakumar, 2021; Pan et al., 2022) and cable-driven (Alamdari and Krovi, 2016; Witte et al., 2017) systems, have been developed and tested for the rehabilitation of stroke survivors. Link-driven exoskeletons are designed to mimic the natural movement of joints using mechanical links. However, such systems can be cumbersome, making training challenging for long periods and causing discomfort to users. On the other hand, cable-driven exoskeletons use cables and motors to achieve joint movement, rendering more lightweight, comfortable, and transparent designs (Jin et al., 2018; Shoaib et al., 2021; Qian et al., 2018), offering easier reconfigurability and reducing the donning on/off time.

In models of the lower limbs, when aided by an exoskeleton, they are often represented as a set of rigid links that consider various inertial parameters (Lyu et al., 2016; Mayag et al., 2022; Gao et al., 2022). Cable-driven exoskeletons utilize rigid-link models of the lower limbs and leverage cable tension to apply joint moments. Studies have demonstrated their considerable promise in rehabilitation (Fang et al., 2021;Jin et al., 2015; Qian et al., 2023; Zhong et al., 2022), as they enable trajectory tracking with low inertia and lightweight designs (Nair and Ezhilarasi, 2020). However, exoskeletons’ design, development, and validation can be costly and time-consuming, often requiring iterative adjustments and experimental tests on human subjects. Model-based simulation studies (Nair and Ezhilarasi, 2020; Rangan et al., 2022; Wang et al., 2020; Varma et al., 2022) offer a viable alternative, providing parametric tools to identify key design parameters and their influence on performance. These studies also provide insights into functional aspects, such as cable tension requirements and the interface between the exoskeleton and the human body, including joint force components. Adjusting cable tension based on the user’s gait phase and impairment level can optimize assistance, encouraging active participation in movement rather than passive support. Joint force components, induced at physiological joints due to cable routing and tension, are critical design variables, as higher forces can lead to increased joint stress and discomfort.

Our prior research (Prasad et al., 2022a, 2022b, 2023a) focused on developing a link-based model of the lower limb and its application toward creating a lower limb cable-driven exoskeleton. We developed a generalized framework for analyzing exoskeletons that use cables to generate motion. The framework was designed to analyze the exoskeleton’s performance in both the sagittal and combined sagittal–frontal planes, accounting for the moments generated by the passive tissues only, that is, neglecting the active contribution of the limb’s muscles. The framework’s modular design allowed changes in the number of cables, routing, cuff placement, subject anthropometric data, reference trajectory, and other parameters that influence tracking performance and design requirements. The framework was employed to identify a suitable configuration of a cable routing-based design of the cable-driven lower limb rehabilitation exoskeleton (C-LREX) that achieves the desired tracking performance with optimal design requirements among several possible configurations. The required cable tension and motor/actuator specifications needed to realize the desired tracking performance were determined. The optimal cuff parameters required for the exoskeleton to function effectively were also predicted. C-LREX (Prasad et al., 2022b,^2023^, ^2024^) is a simulation-based framework for designing and evaluating cable-actuated lower limb rehabilitation devices. Unlike physical prototype-driven approaches, C-LREX utilizes computational modeling to systematically analyze cable routing configurations, ranging from simple planar to biomechanically inspired biplanar architectures. Before physical implementation, this simulation platform enables virtual assessment of critical performance parameters, including trajectory tracking, joint torque profiles, and cable tension requirements. It offers researchers a powerful tool to explore design trade-offs in cable-driven systems, optimizing factors such as energy efficiency, ROM, cable routing architecture, and clinical applicability while avoiding the costly iterative prototyping of conventional exoskeleton development (Prasad et al., 2024).

In our previous work, the C-LREX model tracked reference healthy trajectories without accounting for the active contributions of subjects. This study extends that work by evaluating the model’s ability to correct five distinct stroke gait patterns in the sagittal plane (Kim et al., 2021), incorporating patient-specific anthropometric data and active joint moment contributions. The focus is on assessing the exoskeleton’s capability to correct various levels of gait impairment while adapting to different user anthropometries. In addition, we investigate whether extending the allowable cable tension range can enhance correction for more severe impairments. This study provides a numerical evaluation of the C-LREX configuration’s ability to correct specific levels of impairments for given subjects within defined constraints, serving as a critical step before prototyping and testing.

## 2. Methodology

**Figure 1.**
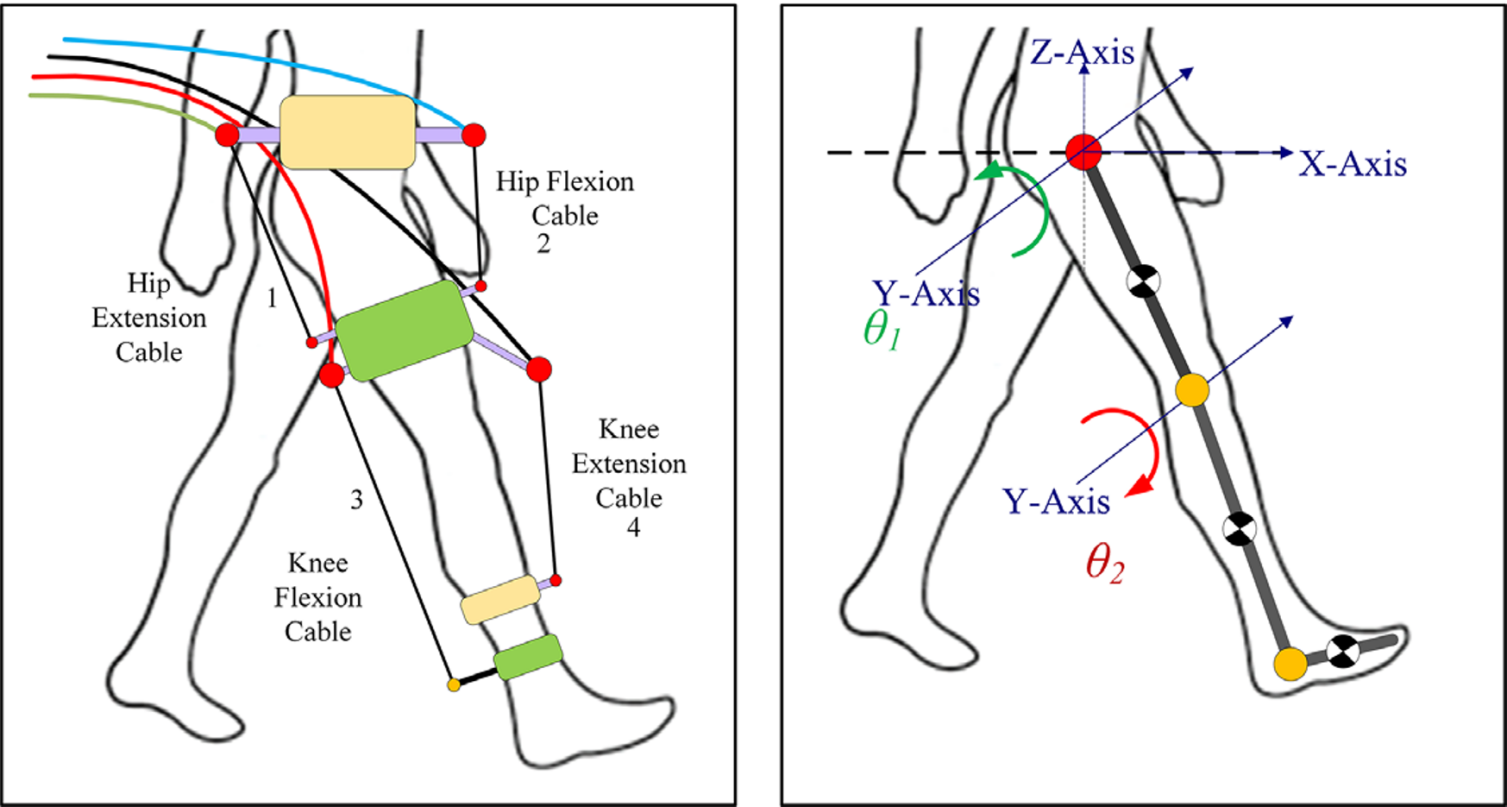
Four cable-driven conceptual models (left) and a lower limb model for C-LREX (right).

In the generalized framework, the lower limb is modeled as a *two-link* pendulum, with one degree of freedom corresponding to flexion/extension at the knee and hip joints, respectively, as shown in Figure 1 (Prasad et al., 2022a, 2022b). The foot was fixed perpendicular to the shank, and the leg was assumed to be suspended in the air. A model with a four-cable configuration, determined previously using the generalized framework, was used. This model demonstrated high overall performance in gait trajectory tracking and satisfied the design requirements.

The equation of motion of the lower limb model was formulated as (Prasad et al., 2022b):(1)

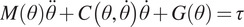

where *M, C*, and *G* are the inertial matrix, Coriolis component, and gravitational components, respectively, while *τ* represents the torques on the joints.

The C-LREX system is primarily designed to provide assistive support during the swing phase of gait, which is often the most impaired phase in post-stroke individuals due to foot drop, reduced toe clearance, and limited joint control (Wang et al., 2020). This design choice is guided by the device’s assistive – not augmentative – nature, targeting individuals who retain partial stance-phase capability. Incorporating full stance-phase support would significantly increase the required joint torques, necessitating larger actuators and stiffer cables, thereby compromising the lightweight and portable design of the system. As such, the current model simplifies stance-phase dynamics by assuming a suspended leg, which allows for a focused evaluation of swing-phase corrections.

**Figure 2.**
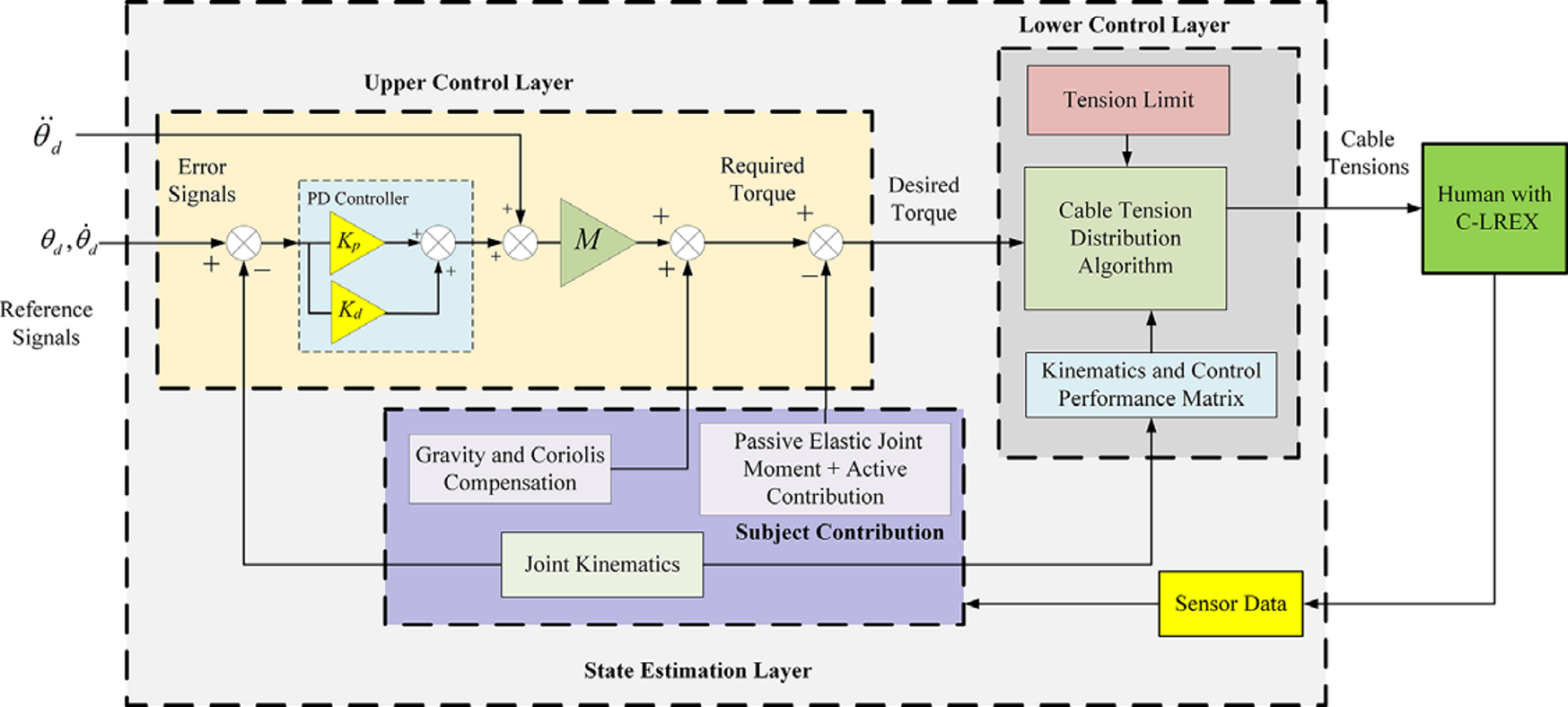
Three-layer control architecture.

The C-LREX system utilized a three-layer control architecture (Prasad et al., 2022) with a proportional-derivative (PD) controller for tracking a reference healthy trajectory, as depicted in Figure 2. The control architecture consisted of an upper control layer, a state estimation layer, and a lower control layer. The upper control layer utilized the PD controller to calculate the required torque based on the difference between the healthy reference and the tracked trajectory. The state estimation layer estimated parameters required by other control layers, such as the subject’s joint moment contributions (both active and passive), the inertial matrix (*M*), the Coriolis matrix (*C*), and the gravitational matrix (*G*) based on joint kinematic data (angular position and velocity). The lower control layer distributed the desired torque among the cables using a quadratic programming approach. This layer ensured that the cable tension met the torque requirements generated by the upper control layer while also satisfying cable tension limits. The dynamic model is implemented in MATLAB® using custom-developed scripts. The system’s equations of motion (Equation 1), representing the coupled human-exoskeleton dynamics, were numerically integrated using a fixed-step fourth-order Runge–Kutta solver with a time step of 0.01 s. The joint torques are computed in real time based on the current system state and cable tension inputs. A previous study (Prasad et al., 2023b) evaluated different controllers, such as PD (Proportional-Derivative) and NMPC (Nonlinear Model Predictive Control), revealing that PD offers faster response times with some performance trade-offs. Due to sampling rate constraints and the computational demands of MPC-based methods, the PD controller was selected for this study.

The subject’s passive contribution to the joint moment is modeled as the passive elastic joint moment induced by the tissues surrounding the joint. This contribution is estimated by using a double exponential equation model, as shown in Equation (2), adopted from the work of Riener and Edrich (1999):(2)

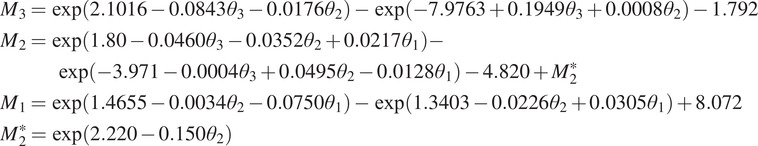

In Equation (2), subscripts 1, 2, and 3 refer to the hip, knee, and ankle, respectively. Since the ankle joint is assumed to be fixed, only hip and knee passive elastic joint moments are considered in the model.

To incorporate the stroke survivor’s actual contribution, the equivalent joint moment (active contribution) corresponding to stroke kinematics is estimated using Equation (1) as shown in Equation (3). It is assumed to be the same for any other gait cycle.(3)

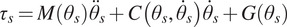

The total subject contribution during trajectory tracking was assumed to be the sum of the passive elastic joint moment and the moment estimated using the rigid link model (Equation [2]), as shown in Equation (4).(4)

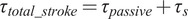



**Figure 3.**
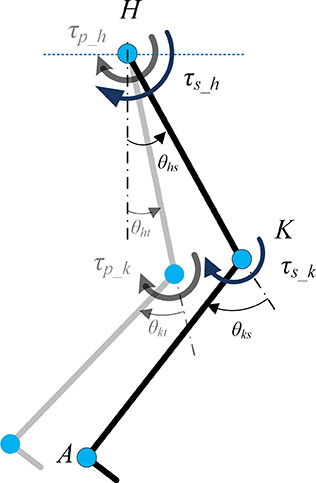
Stroke and reference limb position and corresponding inputs (subscripts s and t refer to stroke and model-tracked case, *p* refer to passive, and h and *k* refer to the hip and knee joint, respectively).

The passive elastic joint moment was calculated based on the joint angle of the reference trajectory for the entire limb. The calculation was adjusted if the model-tracked angle deviated from the reference angle. The passive moment was calculated based on the final tracked angle to account for the overall passive contribution, as illustrated in Figure 3.

### 2.1. Anthropometry and trajectory

This study utilized the dataset published by Fukuchi et al. (2018) to establish a reference healthy trajectory. A slower gait (more than 30% slower than the average comfortable speed) with a gait cycle time of 3.48 s was selected as the reference healthy trajectory for tracking, imitating the typical slow walking speed of stroke survivors. The stroke gait trajectory time was also assumed to be the same (3.48 s) for tracking analysis purposes. The kinematic data of five different stroke gait patients, called stroke gait SG1–5, were obtained from the clustered gait data published by Kim et al. (2021). The dataset was collected from 36 stroke patients over 6 months, during which six different clusters were identified through clustering and classification from 111 gait cycles.

**Figure 4.**
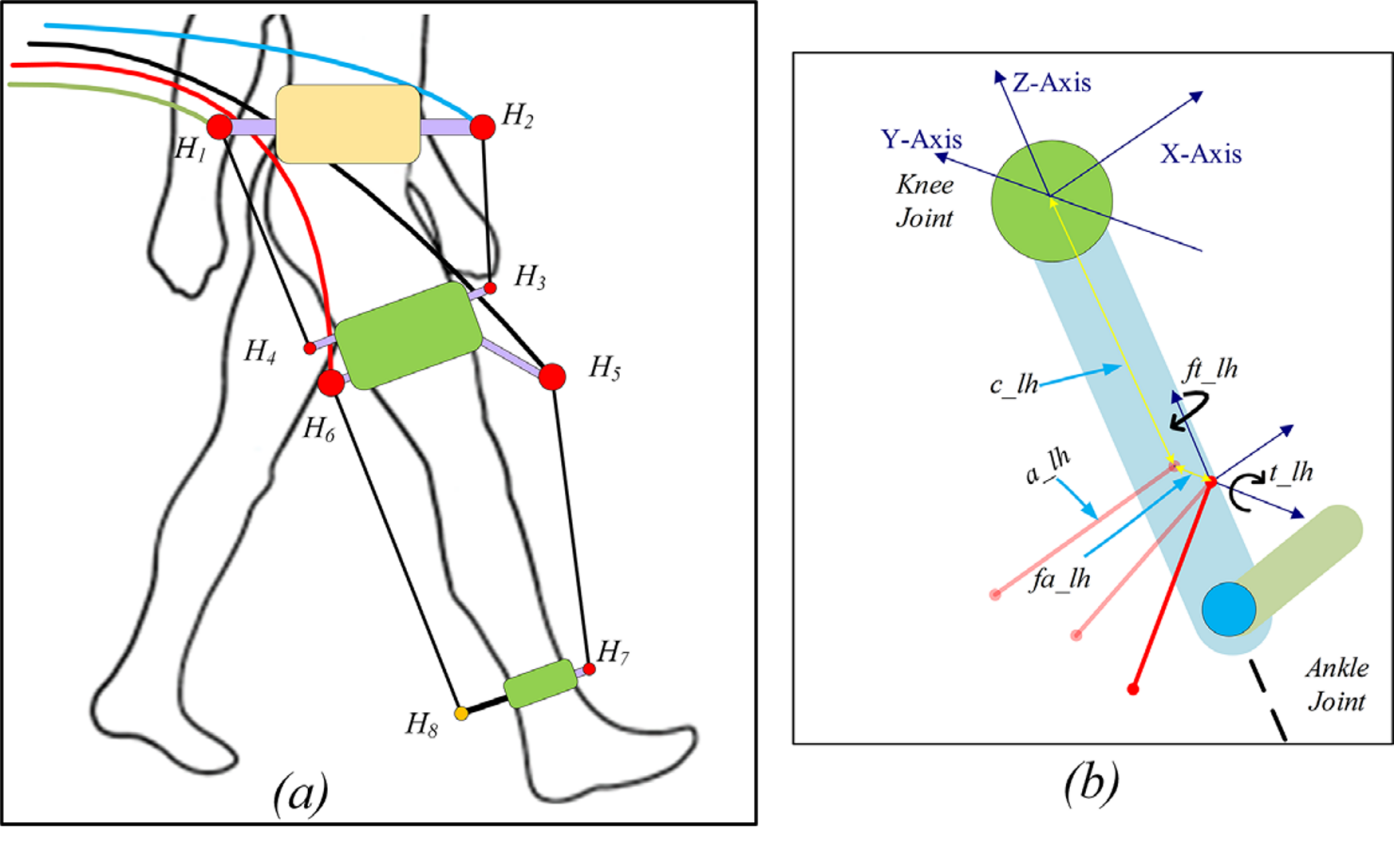
Conceptual configuration of C-LREX with hinges and cables (a) and definition of hinges with respect to the nearby joint using a five-parameter definition (Prasad et al., 2023a) (b).

Anthropometric data (mass, length, and moment of inertia of limb segments) were calculated from the subject’s height and mass based on Winter’s model (Winter, 2009). These data are used to scale the cable-driven exoskeleton and estimate the coefficients in Equations (1)–(3). It was assumed, however, that the impaired leg could achieve the ROM of a healthy leg, disregarding the potential effect of muscle spasticity. The influence of the level of impairment and the correction required for the impaired gait patterns were simulated based on one subject (height and mass of 179.3 cm and 78.5 kg, respectively) selected from the Fukuchi dataset (Fukuchi et al., 2018). The cuff’s distance from the physiological joint center is defined in proportion to the limb segment lengths (apart from the hip level cuff) employing five parameter-based definitions, as shown in Figure 4. The thigh cuffs are set at 65 and 75% of the thigh length, while the shank cuffs are set at 75%. The cuff dimension for the base subject is listed in Table 1.

**Table 1.** Cuff definition parameters

Hinge number	Cuff parameters					Joint
	Length (m)			Angle (deg)		
	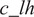	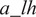	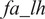	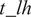	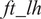	
H_1_	0.0500	0.2000	0	180	0	Hip
H_2_	0.0500	0.2000	0	0	0	Hip
H_3_	−0.2820	0.1500	0	0	0	Knee
H_4_	−0.2820	0.1500	0	180	0	Knee
H_5_	−0.3254	0.2000	0	60	0	Knee
H_6_	−0.3254	0.1500	0	0	0	Knee
H_7_	−0.3295	0.1000	0	0	0	Knee
H_8_	−0.3295	0.1000	0	180	0	Knee

The influence of anthropometry variation on the correction of gait impairment was accomplished based on data from 6 subjects with variant anthropometric data. The implementation ignores cuff inertial data and only considers biological limb-segment inertia information. However, the model’s parametric structure allows for the future incorporation of combined limb-cuff inertial properties (by superimposing cuff inertia tensors onto existing limb segment parameters.

To assist the impaired limb in following the reference trajectory, the C-LREX model exerted joint moments via cable tension. The cable tension range was limited to 7–100 N (consistent with prior work (Alamdari and Krovi, 2016; Jin, 2018)) to ensure that the cable remained taut during operation and the tension remained within a safe range for use as an assistive device.

## 3. Simulation results and discussion

The model was simulated by implementing the framework, and the tracked trajectories, cable tension, and ankle position tracking were analyzed.

### 3.1. Influence of impairment level on gait correction

**Figure 5.**
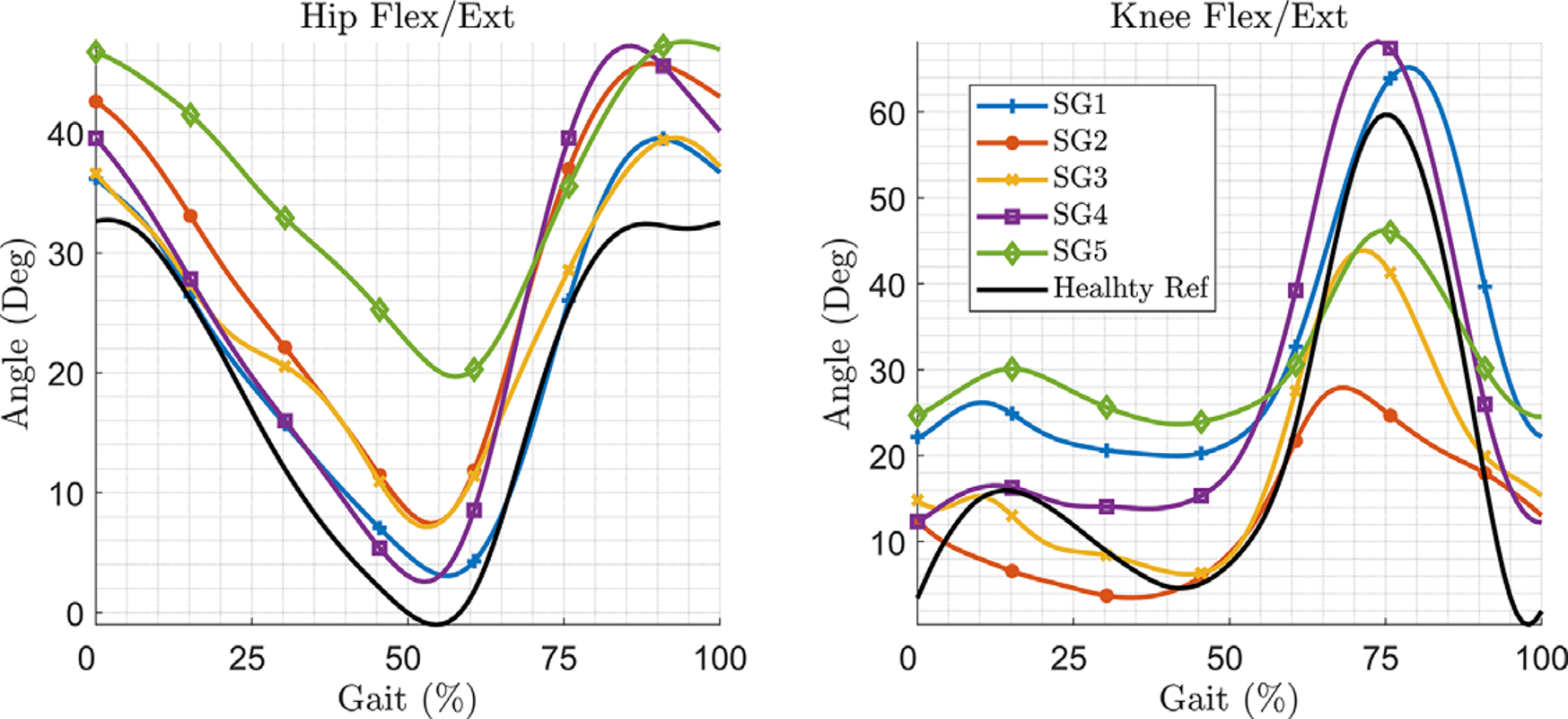
The healthy and impaired gait employed in the study (SG refers to stroke gait).

The five impaired stroke gait patterns, referred to as SG, and the reference healthy gait included in the study are shown in Figure 5. SG5 had the highest deviation from the reference healthy gait (~20° deviation in the hip joint during 30–60% of the gait cycle).

**Figure 6.**
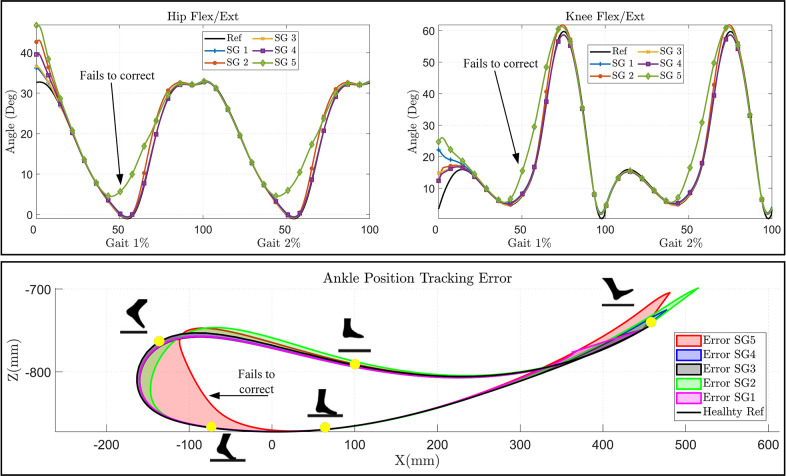
Joint angle tracking (for two gait cycles) (top) and error in ankle position tracking (bottom) for various impaired gaits (shown for one gait cycle).

The joint angle and ankle position tracking are depicted in Figure 6. The ankle’s position was measured by taking the hip joint as a reference point. The error in ankle position tracking, representing the deviation of the model-tracked trajectory from the reference trajectory, was estimated for all five stroke gait patterns. The C-LREX model was able to correct SG1–4 successfully, while for SG5, the correction was not as successful, and the tracking error reached up to 10° in 55–65% of gait cycles.

The initial deviations seen in the hip and knee joint angles in the C-LREX model from the healthy trajectory (Figure 6) are likely due to the starting boundary conditions, where the model starts from the initial position of the stroke gait (altered ROM of the hip and knee). However, these initial deviations decrease rapidly and vanish by the second gait cycle.

**Figure 7.**
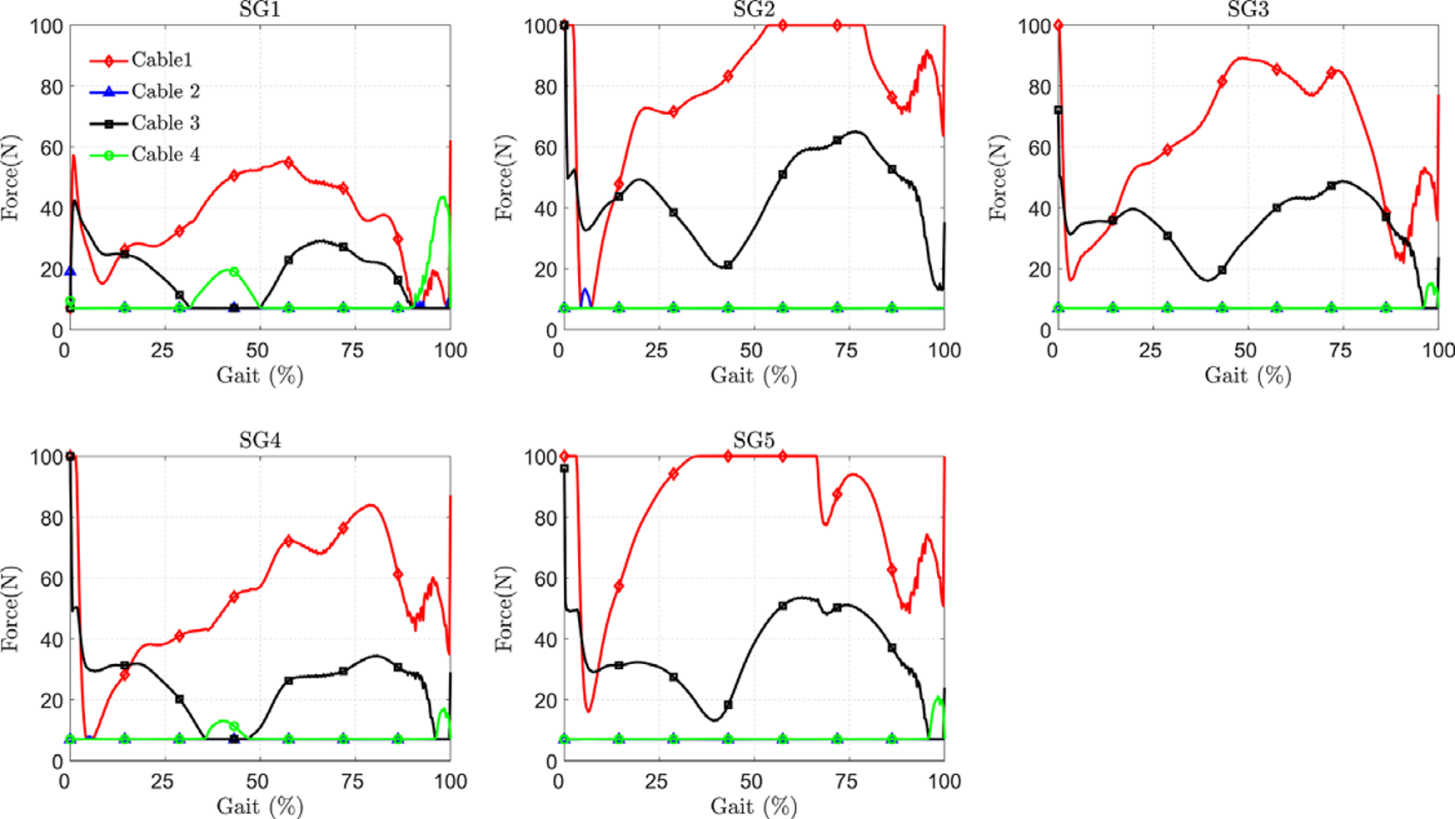
Cable tension requirements in correcting different impaired gaits (shown for one gait cycle).

The required cable tension in the C-LREX model for the impaired gait correction is shown in Figure 7. Unlike our previous work (Prasad et al., 2022a, 2022b), where all four cables were activated during trajectory tracking, the hip flexion and knee extension cables were not actively engaged in this study (assigned only the minimum tension to keep the cables taut), as shown in Figure 7. This is due to the inclusion of the subject’s active contribution (stroke gait contribution) during tracking, along with the passive elastic joint moment. However, the actual active contribution of a stroke patient may vary due to specific muscle impairment, which is not considered in the current simplified model.

The results demonstrated that the limited range of cable tension prevented the C-LREX model from generating the required joint moment during the 60–80% cycle of SG2 and the 35–70% cycle of SG5, thereby failing to closely track the healthy reference trajectory. Although the knee cable tension remained within the specified limit, the C-LREX model could not closely follow the knee trajectory in the SG2 and SG5 patterns since applying the knee extension moment would increase the requirements of the hip flexion moment.

**Figure 8.**
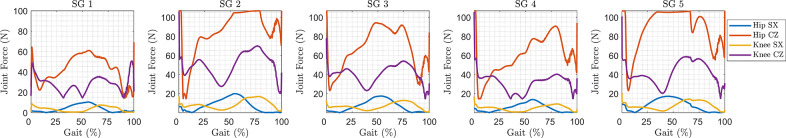
Joint force components induced at the hip and knee joints due to applied cable tension (SG refers to stroke gait) (shown for one gait cycle).

The force components (SX: anterior–posterior shear force in the X direction and CZ: compressive force in the Z direction) induced at the hip and knee joints due to the applied cable tension in the C-LREX model are plotted in Figure 8. The model closely tracked the healthy reference trajectory for SG1, resulting in the lowest joint force components and, consequently, the least stress and discomfort for the user. In contrast, the joint force components remained higher for a longer duration in SG5, which may lead to more significant discomfort for the subject as the impairment level exceeds the system’s correction capabilities.

### 3.2. Influence of subject anthropometric variability on gait correction

In the previous sections, we evaluated the effectiveness of the C-LREX model in correcting various stroke gait patterns with different levels of impairment. In this section, we present an investigation of yet another critical factor, the user’s limb anthropometry, which could impact the model’s tracking performance. Specifically, we assessed the model’s ability to correct impaired gait patterns in patients with varying anthropometric characteristics. Toward this, anthropometric data were obtained for 42 subjects from the Fukuchi dataset, including estimating their body mass index (BMI) values. The subjects were classified into two categories (Table 2): normal weight and overweight, which included underweight (BMI *≤* 25 kg/m^2^) and obese subjects (BMI > 25 kg/m^2^), respectively, to account for uneven data distribution across BMI ranges. We calculated the mean and standard deviation (SD) values to ensure equal representation of subjects. We selected three subjects with BMI values close to the mean or within 1 SD of the mean (mean and mean ± SD) for each weight category. The anthropometric information of these selected subjects is presented in Table 3.

**Table 2.** BMI ranges and distribution of the subjects

Class	Ranges (BMI) (kg/m^2^)	Male subject	Female subject	Sum	Mean BMI	SD BMI
Normal	≤25	12	12	24	21.496	2.0199
Overweight	>25	12	6	18	28.111	2.2987
Total		24	18	42

**Table 3.** Selected subject anthropometric information

Category	Anthropometry	Subject 1	Subject 2	Subject 3
Normal	Mass (kg)	48.80	66.25	61.70
	Height (cm)	158.0	175.8	162.2
	BMI (kg/m^2^)	19.476	21.496	23.516
Overweight	Mass (kg)	70.50	79.65	70.55
	Height (cm)	164.5	168.2	149.7
	BMI (kg/m^2^)	25.813	28.111	30.410

**Figure 9.**
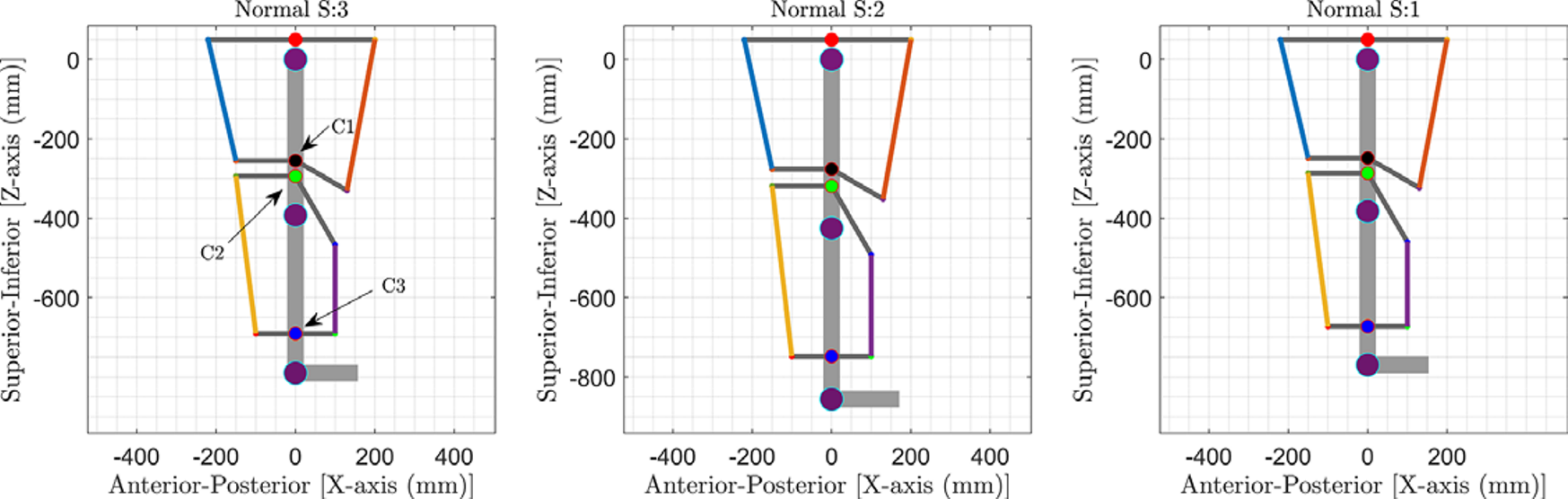
Cuff locations in the models of the three subjects (S refers to subjects).

The C-LREX model was personalized for the lower limbs of the selected subjects by scaling the model and calculating the inertial properties (segment mass, length, and inertia) of the limbs based on the subjects’ mass and height values, using the Winter model (Winter, 2009). In addition, to maintain consistent cable routing in the C-LREX model for all subjects, the distance of the cuffs was scaled only along the limb axis according to the user’s anthropometric data (Figure 9). The distance of the cuff from the nearest physiological joint along the limb section was defined as a proportion of the limb segment length for the cuffs on the thigh and shank zones. In Figure 9, the distance of cuffs C1 and C2 from the hip joint was kept at the same proportion (65 and 75% of the thigh length, respectively) for all the subjects. Similarly, the distance of cuff C3 from the knee joint was kept at the same proportion (75% of the shank length). However, the cuff lengths remained the same for all three subjects.

**Figure 10.**
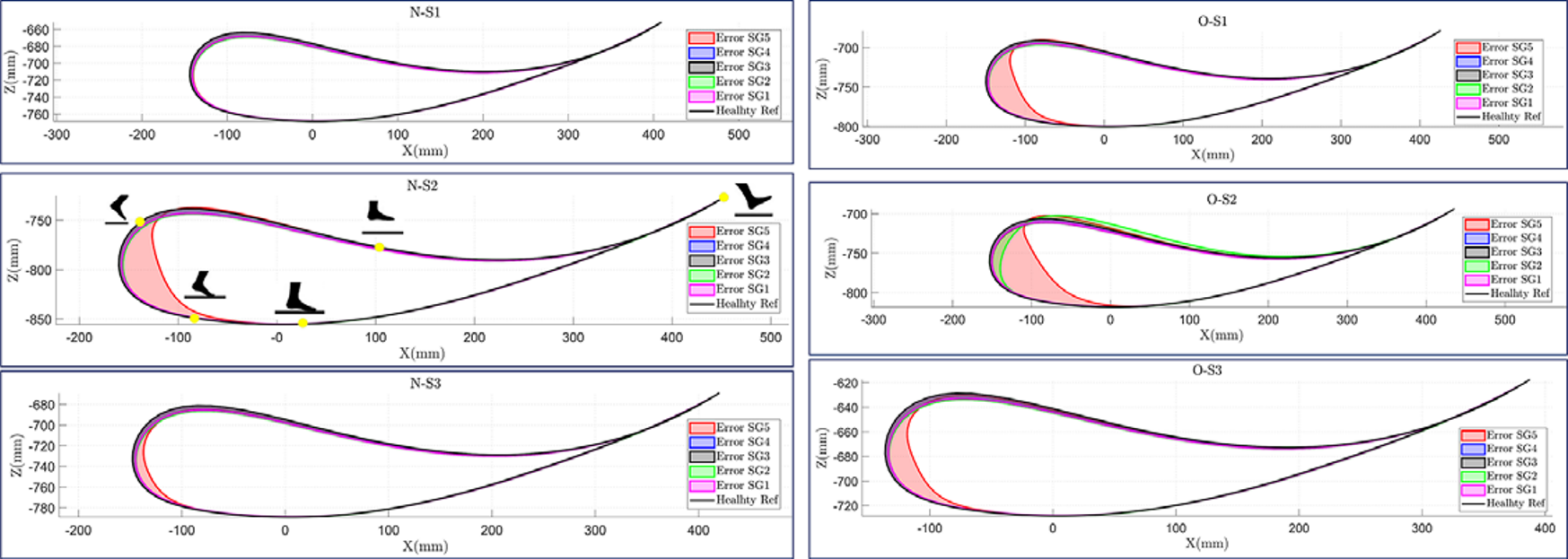
Trajectory tracking with different subjects for different impaired gaits (SG refers to stroke gait; N and O refer to normal and overweight class, respectively, and S1, S2, and S3 correspond to Subjects 1, 2, and 3 of class).

Figure 10 shows the ankle position tracking error for the hip and knee joint angles of five different impaired gait patterns simulated by the C-LREX model for each subject. The results show that the impairment level was the most influential parameter that increased the C-LREX model’s tracking error. SG5, with the highest impairment level, caused noticeable tracking errors (during 40–70% of the gait cycle corresponding to the pre-swing to mid-swing phase) for all subjects, except for one normal-weight (N-S1) subject. Anthropometric variations had a minimal impact on tracking performance, as cuff locations were adjusted according to each subject’s lower limb anthropometry to maintain consistent limb segment proportions. In addition, variations in limb segmental mass, height, moment of inertia, and the coefficients in Equations (1) and (3) (*M*, *C*, and *G* matrices), along with the subject’s active and passive contributions during motion, influenced the system dynamics. These collective variations resulted in similar tracking errors across different anthropometric profiles for a given stroke gait pattern.

The distance between the ankle and the hip joints varied based on the subject’s anthropometry. The root mean square error (RMSE) was used to quantify the model’s error in tracking the healthy trajectory, and it was found to vary with the subject’s BMI and impairment level. The highest RMSE was observed in overweight stroke gait SG5, which had a significant impairment level. A two-way analysis of variance (ANOVA) test was conducted to further evaluate the combined effects of user anthropometry and impairment level on the RMSE values of the ankle joint position tracking. The results of the test are presented in Table 4.

**Table 4.** Two-way ANOVA analysis result of ankle position tracking

Source	Sum of squares	DF	Mean square	*F* value	*p* value
Impairment level (SG)	5.62E−05	4	1.40E−05	10.44733	9.80E–05
Anthropometry (BMI)	1.58E−06	1	1.58E−06	1.172159	.291839
Interaction	5.94E−06	4	1.48E−06	1.10462	.381716

**Figure 11.**
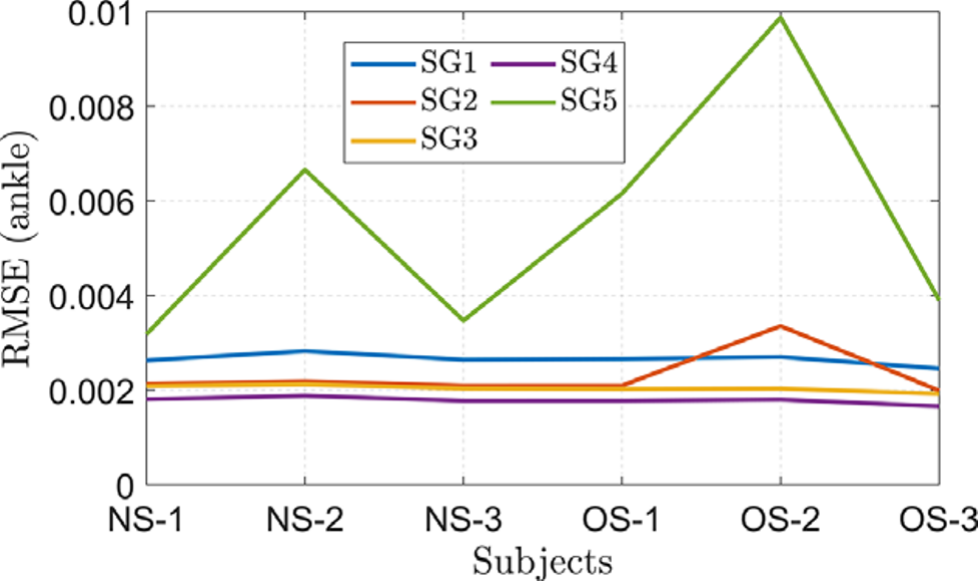
Interaction plot of subjects and stroke gait: NS and OS refer to normal and overweight subjects, respectively.

**Figure 12.**
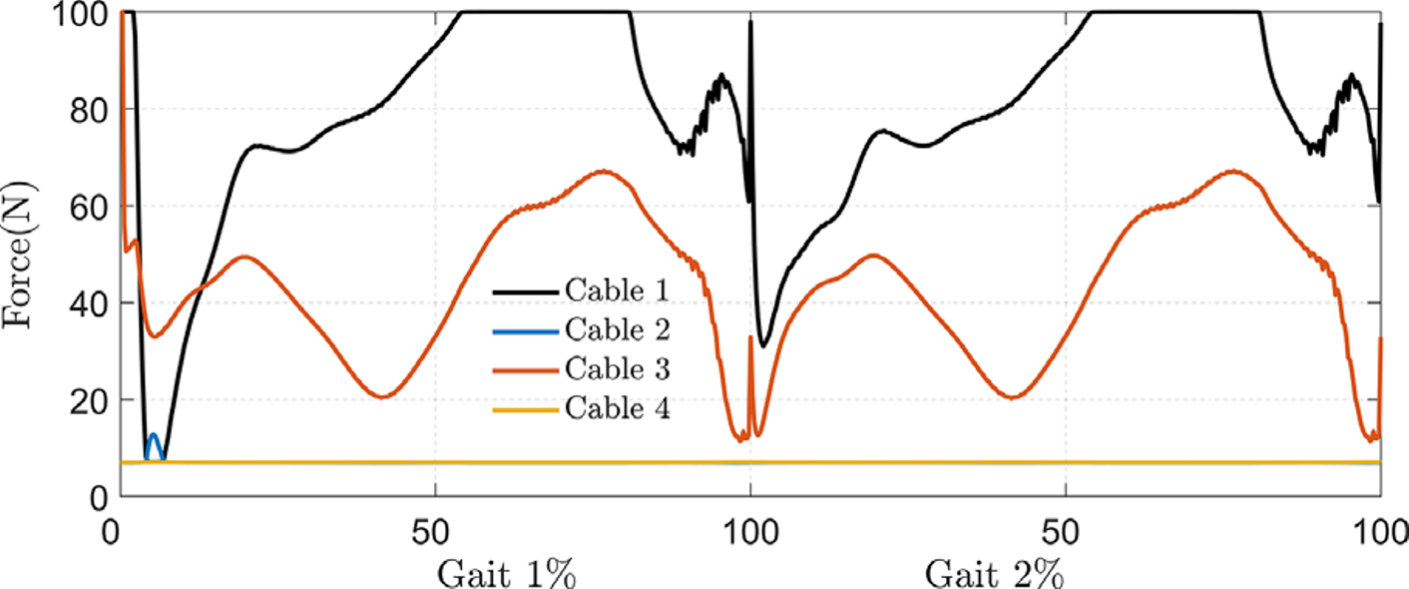
Cable tension requirement in subject OS-2.

According to the ANOVA results, impairment level significantly affects the overall tracking performance, whereas user anthropometry (BMI) does not. In addition, the interaction between stroke gait and user anthropometry was insignificant, as shown in the interaction plot (Figure 11). Furthermore, irrespective of the user’s BMI, the C-LREX model successfully tracked the ankle trajectory with similar RMSE for SG1–4 (with minor variation in SG2 for the obese subject OS-2, where the cable tension reached the maximum allowable limit during a small percentage of the gait cycle, as shown in Figure 12) confirming BMI-independent correction. Since the model failed to track SG5 closely (for each subject except N–S1, the cable tension required was higher than the allowed maximum limit, similar to the cable tension observed for SG5 in Figure 7), the RMSE kept varying randomly for SG5 gait.

The results confirmed that the C-LREX model could assist different levels of impaired gait for users with various BMI ranges as long as the required cable tension remains within the specified range. However, the model does not account for any physiological conditions associated with the pathology, such as muscle weakness, stiffness deterioration, or spasticity. The framework is open to incorporating such information if a quantitative model exists. The results estimate the maximum cable tension, which is essential for determining actuator specifications. After scaling the cuff location along the limb axis-based subject-specific anthropometric data, the model reestimated the joint moment using the same applied cable tension. The insensitivity of the model’s tracking performance to subject anthropometry is an essential aspect of the C-LREX technology, making it more practical and usable for a broader population of stroke survivors with minimal modifications. However, it is vital to note that the results were based on an ideal scenario, where the C-LREX model corrects the stroke trajectory to match or get as close as possible to the reference trajectory in a single gait cycle. In reality, a lower limb exoskeleton would assist the user in progressively correcting the impaired gait and approaching/reaching the healthy trajectory throughout training.

### 3.3. Correcting SG5 with extended cable tension range

**Figure 13.**
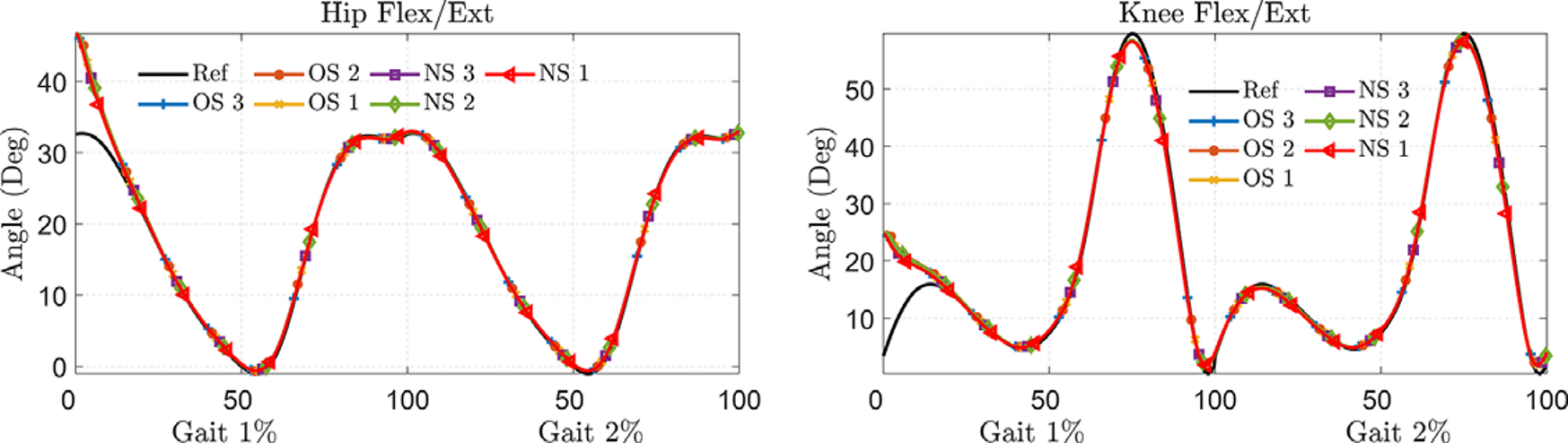
Trajectory correction for SG5 gait with different subjects (for extended cable tension range to 150 N).

As discussed in the previous section, the current model failed to track closely the reference healthy trajectory for SG5 due to cable tension restrictions. Due to higher deviation in the hip ROM compared to other gait impairments, SG5 demanded a higher joint moment, which the C-LREX model failed to provide due to limitations on the applicable cable tension range. We extended the maximum applicable cable tension range to 150 N to confirm this scenario. We simulated the C-LREX model to correct the stroke gait pattern SG5 for the six different subjects studied in the previous section. Indeed, the simulation results (as depicted in Figure 13) indicated that extending the cable tension range corrected the SG5 gait impairment once the cable tension constraint was relaxed.

Depending on the level of gait impairment, cable tension can be treated as a design parameter with a variable range of applicable values (7–100 N or higher). The variation in cable tension directly correlates with actuator requirements and indirectly with the induction joint force components. A Monte Carlo-based optimization approach (e.g., a weighted sum optimization function of cable tension and joint force components) (Prasad et al., 2024) can be employed to estimate the optimal cable tension limit that corrects the impairment while minimizing joint force exertion.

While considering a fixed foot in our study may not be entirely accurate, it served as a practical assumption for analyzing C-LREX’s design performance and identifying optimal cable routing configurations, particularly considering that C-LREX primarily assisted in the swing phase of gait. Future models could benefit from expanding the model to include the foot dynamics. On the other hand, the current model demonstrated the capability to correct various patterns of impaired stroke gait (up to a 20° deviation in the hip’s ROM when the cable tension range was extended). Future work could also benefit from real-time joint kinematic data and muscle electromyography data, reflecting a more realistic active contribution of the user’s lower limb muscles, which may change during gait and the training period.

Our dynamic model effectively captures the primary torque impairments associated with post-stroke gait, as represented in the Equation 4. However, it includes several physiological simplifications. First, the model does not incorporate the velocity-dependent characteristics of spasticity (Fujimura et al., 2022), which clinically manifests as hyperreflexia and time-varying joint resistance. This limitation may affect the accuracy of swing-phase simulations, particularly during terminal swing, when spasticity typically peaks. Second, the model assumes constant impairment levels, which overlooks critical factors such as task-dependent muscle weakness and fatigue-induced torque decay, which can significantly diminish muscular output during prolonged walking (Knarr et al., 2013).

Notably, while the dataset includes data from multiple subjects, it may not fully capture the diversity of anthropometric variations and gait impairments observed in the broader stroke survivor population. In addition, while the simulation framework accounts for key biomechanical parameters, it does not fully replicate the complexities of human muscle–tendon dynamics or soft tissue deformations. These simplifications could introduce biases when extrapolating simulation results to real-world scenarios. Furthermore, potential errors or artifacts in signal processing could also lead to mismatches between the exoskeleton’s assistance provided and the actual requirements of the user. These limitations highlight the need for further validation with more extensive and diverse datasets and experimental testing to confirm the simulation’s practical applicability. In future work, sensor noise and uncertainty can be explicitly modeled to simulate real-world disturbances, enabling a more realistic system performance evaluation. In addition, incorporating robust control strategies (adaptive control) may enhance the system’s resilience to signal variability and improve its effectiveness in real-world scenarios.

The current study demonstrated the potential of the C-LREX in correcting impaired stroke gait and estimated the required cable tension range for the actuators to achieve the desired trajectory. The C-LREX offers a promising design solution that can be adapted to various patients with different levels and types of impaired gait by adjusting key parameters. The simulation-based model presented in this work provides a cost- and time-effective quantitative platform for data-driven design and fabrication processes. It delivers all the necessary information for the successful implementation, including cable tension ranges (actuator capacity), routing details, and cuff locations, rendering it inherently suited for specific gait impairments and patient-specific needs. Proper incorporation of the specific physiological deficits associated with impaired gait should be considered carefully in conjunction with an expert clinical team to improve the model’s accuracy. Such simulations can provide valuable insight for researchers and engineers developing cable-driven exoskeletons for clinical assistive purposes. Future work will include incorporating hip abduction and adduction in the frontal plane to address gait impairments such as circumduction gait. Ongoing and future efforts will expand the current simulation model to include multiple bi-planar stroke gait trajectories and prototype the exoskeleton to correct such bi-planar impairments at varying dysfunction levels. Clinical studies targeting patient-specific gait adaptations and specific impairment levels are also planned to validate the exoskeleton’s effectiveness and facilitate broader clinical adoption. The current model’s assistance is limited to the swing phase of gait, which reflects a deliberate design choice aligned with the clinical profile of many post-stroke individuals who retain partial stance-phase capability. While the swing phase typically exhibits the most pronounced kinematic deviations in post-stroke gait, this assumption inherently limits the system’s applicability to individuals with more severe impairments requiring support throughout the gait cycle. Future research should extend the C-LREX framework to include the stance-phase dynamics through hybrid control strategies that adaptively switch or blend support modes based on real-time gait phase detection. Such an approach will allow for comprehensive gait assistance and broaden the potential user population, especially in cases involving asymmetric stance control, fatigue, or balance deficits. In addition, incorporating ground interaction models and variable impedance control during the stance phase could enhance safety, stability, and neuromuscular engagement during rehabilitation.

## 4. Conclusions and future work

Cable-driven exoskeletons offer several advantages over traditional link-driven exoskeletons, including lightweight, negligible inertia, exerting inertial vibration, and having easier donning on/off. This study employed a four-cable-driven model of C-LREX to investigate the correction of impaired stroke gait. While the model successfully corrected the impaired gait to track a healthy reference trajectory, the impairment level significantly impacted trajectory tracking. The impaired stroke gait pattern with a higher hip ROM (>20°) failed to track the reference healthy trajectory closely due to predefined cable tension ranges. Still, it was mitigated by identifying the cable tension requirements needed to correct the impairment. The variation in user anthropometry had a minimal impact due to the scaling of cuffs along the limb axis.

Overall, the findings suggest that C-LREX has the potential for correcting impaired gait patterns and can be adapted to various types and levels of gait impairment and, thus, to a broader subject population. The analysis used a simulation-based approach and will require proper validation through experimental testing. Furthermore, the model is currently limited to sagittal plane motion tracking. Future work will incorporate hip joint abduction and adduction in the frontal plane to address gait impairments such as circumductory motion, which is common in post-stroke patients. The model will be simulated with multiple biplanar stroke gait and then prototyped to correct bi-planar impairment with a specific level of impairment.

## Data Availability

The data supporting the findings of this study are available within the article and/or its Supplementary Materials. The data used in the study will be made available upon reasonable request.

## References

[r1] Alamdari A and Krovi V (2016) Design and analysis of a cable-driven articulated rehabilitation system for gait training. Journal of Mechanisms and Robotics 8(5). 10.1115/1.4032274.

[r2] Balaban B and Tok F (2014) Gait disturbances in patients with stroke. PM&R 6(7), 635–642. 10.1016/j.pmrj.2013.12.017. 24451335

[r3] Fang J, Haldimann M, Marchal-Crespo L and Hunt KJ (2021) Development of an active cable-driven, force-controlled robotic system for walking rehabilitation. Frontiers in Neurorobotics 15(May), 1–16. 10.3389/fnbot.2021.651177. PMC8176959 34093158

[r4] Feigin VL, Brainin M, Norrving B, Martins S, Sacco RL, Hacke W, Fisher M, Pandian J and Lindsay P (2022) World stroke organization (WSO): Global stroke fact sheet 2022. International Journal of Stroke 17(1), 18–29. 10.1177/17474930211065917. 34986727

[r5] Fujimura K, Mukaino M, Itoh S, Miwa H, Itoh R, Narukawa D, Tanikawa H, Kanada Y, Saitoh E and Otaka Y (2022) Requirements for eliciting a spastic response with passive joint movements and the influence of velocity on response patterns: An experimental study of velocity-response relationships in mild spasticity with repeated-measures analysis. Frontiers in Neurology 13, 1–9. 10.3389/fneur.2022.854125. PMC9007406 35432169

[r6] Fukuchi CA, Fukuchi RK and Duarte M (2018) A public dataset of overground and treadmill walking kinematics and kinetics in healthy individuals. PeerJ 6(4), e4640. 10.7717/peerj.4640. 29707431 PMC5922232

[r7] Gao M, Wang Z, Pang Z, Sun J, Li J, Li S and Zhang H (2022) Electrically driven lower limb exoskeleton rehabilitation robot based on anthropomorphic design. Machines 10(4), 266. 10.3390/machines10040266.

[r8] Jin X (2018) A Novel Design of a Cable-Driven Active leg Exoskeleton (C-ALEX) and Gait Training with Human Subjects. PhD thesis of Columbia University, School of Arts and Sciences.

[r9] Jin X, Cui X and Agrawal SK (2015) Design of a cable-driven active leg exoskeleton (C-ALEX) and gait training experiments with human subjects. In 2015 IEEE International Conference on Robotics and Automation (ICRA), 2015, pp. 5578–5583, 10.1109/ICRA.2015.7139979.

[r10] Jin X, Prado A and Agrawal SK (2018) Retraining of human gait - are lightweight cable-driven leg exoskeleton designs effective? IEEE Transactions on Neural Systems and Rehabilitation Engineering 26(4), 847–855. 10.1109/TNSRE.2018.2815656. 29641389

[r11] Johnson W, Onuma O, Owolabi M and Sachdev S (2016) Stroke: A global response is needed. Bulletin of the World Health Organization 94(9), 634–634A. 10.2471/BLT.16.181636. 27708464 PMC5034645

[r12] Kim H, Kim Y-H, Kim S-J and Choi M-T (2021) Pathological gait clustering in post-stroke patients using motion capture data. Gait & Posture 94, 210–216. 10.1016/j.gaitpost.2022.03.007. 35367849

[r13] Knarr BA, Kesar TM, Reisman DS, Binder-Macleod SA and Higginson JS (2013) Changes in the activation and function of the ankle plantar flexor muscles due to gait retraining in chronic stroke survivors. Journal of Neuroengineering and Rehabilitation 10(1), 12. 10.1186/1743-0003-10-12. 23369530 PMC3565909

[r14] Lyu M, Chen W, Ding X, Wang J, Bai S and Ren H (2016) Design of a biologically inspired lower limb exoskeleton for human gait rehabilitation. The Review of Scientific Instruments 87(10), 104301. 10.1063/1.4964136. 27802730

[r15] Mayag LJA, Múnera M and Cifuentes CA (2022) Human-in-the-loop control for AGoRA unilateral lower-limb exoskeleton. Journal of Intelligent and Robotic Systems 104(1), 3. 10.1007/s10846-021-01487-y.

[r16] Nair AS and Ezhilarasi D (2020) Performance analysis of super twisting sliding mode controller by ADAMS–MATLAB co-simulation in lower extremity exoskeleton. International Journal of Precision Engineering and Manufacturing-Technology 7(3), 743–754. 10.1007/s40684-020-00202-w.

[r17] Pan C-T, Lee M-C, Huang J-S, Chang C-C, Hoe Z-Y and Li K-M (2022) Active assistive design and multiaxis self-tuning control of a novel lower limb rehabilitation exoskeleton. Machines 10(5), 318. 10.3390/machines10050318.

[r18] Prasad R, El-Rich M, Awad MI, Agrawal SK and Khalaf K (2023a) Bi-planar trajectory tracking with a novel 3DOF cable driven lower limb rehabilitation exoskeleton (C-LREX). Sensors 23, 1677. 10.3390/s23031677. 36772715 PMC9920627

[r19] Prasad R, El-Rich M, Awad MI, Agrawal SK and Khalaf K (2024) Muscle-inspired bi-planar cable routing: A novel framework for designing cable driven lower limb rehabilitation exoskeletons (C-LREX). Scientific Reports 14(1), 5158. 10.1038/s41598-024-55785-0. 38431744 PMC10908813

[r20] Prasad R, Khalaf K, Awad MI and El-Rich M (2023b) Influence of controller on cable driven lower limb rehabilitation exoskeleton (C-LREX): PD vs MPC. In 9th International Conference on Control, Decision and Information Technologies (CoDIT), 6.

[r21] Prasad R, Khalaf K, Awad MI, Hussian I, Jelinek HF, Huzaifa U and Rich ME (2022a) A generalized framework for the assessment of various configurations of cable-driven Mobile lower limb rehabilitation exoskeletons. In Proceedings of the 12th International Conference on Biomedical Engineering and Technology, April, pp.133–140, 10.1145/3535694.3535716.

[r22] Prasad R, El-Rich M, Awad MI, Hussain I, Jelinek HF, Huzaifa U and Khalaf K (2022b) A framework for determining the performance and requirements of cable-driven Mobile lower limb rehabilitation exoskeletons. Frontiers in Bioengineering and Biotechnology 10. 10.3389/fbioe.2022.920462. PMC9251017 35795162

[r23] Qian S, Zi B, Shang W-W and Xu Q-S (2018) A review on cable-driven parallel robots. Chinese Journal of Mechanical Engineering 31(1), 66. 10.1186/s10033-018-0267-9.

[r24] Qian W, Liao J, Lu L, Ai L, Li M, Xiao X and Guo Z (2023) CURER: A lightweight cable-driven compliant upper limb rehabilitation exoskeleton robot. IEEE/ASME Transactions on Mechatronics 28(3), 1730–1741. 10.1109/TMECH.2022.3224423.

[r25] Rangan RP, Maheswari C, Vaisali S, Sriram K, Stonier AA, Peter G and Ganji V (2022) Design, development and model analysis of lower extremity Exo-skeleton. Medical Engineering & Physics 106(June), 103830. 10.1016/j.medengphy.2022.103830. 35926951

[r26] Riener R and Edrich T (1999) Identification of passive elastic joint moments in the lower extremities Journal of Biomechanics 32(5), 539–544, 10.1016/s0021-9290(99)00009-3. 10327008

[r27] Sheffler LR and Chae J (2015) Hemiparetic gait. Physical Medicine and Rehabilitation Clinics of North America 26(4), 611–623. /10.1016/j.pmr.2015.06.006. 26522901

[r28] Shoaib M, Asadi E, Cheong J and Bab-Hadiashar A (2021) Cable driven rehabilitation robots: Comparison of applications and control strategies. IEEE Access 9(August), 110396–110420. 10.1109/ACCESS.2021.3102107.

[r29] Ullas U and Rajendrakumar PK (2021) Design of a low-cost lower limb rehabilitation exoskeleton system. IOP Conference Series: Materials Science and Engineering 1132(1), 012008. 10.1088/1757-899X/1132/1/012008.

[r30] Varma VS, Rao RY, Vundavilli PR, Pandit MK and Budarapu PR (2022) A machine learning-based approach for the design of lower limb exoskeleton. International Journal of Computational Methods 19(08), 1–22. 10.1142/S0219876221420123.

[r31] Wang Y, Liu Z, Zhu L, Li X and Wang H (2020) An impedance control method of lower limb exoskeleton rehabilitation robot based on predicted forward dynamics. In 2020 IEEE 19th International Conference on Trust, Security and Privacy in Computing and Communications (TrustCom), pp. 1515–1518. 10.1109/TrustCom50675.2020.00206.

[r32] Wang Y, Mukaino M, Ohtsuka K, Otaka Y, Tanikawa H, Matsuda F, Tsuchiyama K, Yamada J and Saitoh E (2020) Gait characteristics of post-stroke hemiparetic patients with different walking speeds. International Journal of Rehabilitation Research 43(1), 69–75. 10.1097/MRR.0000000000000391. 31855899 PMC7028468

[r33] Winter DA (2009) Biomechanics and Motor Control of Human Movement, Vol. 2. Hoboken, NJ: John Wiley & Sons, Inc

[r34] Witte KA, Fatschel AM and Collins SH (2017) Design of a lightweight, tethered, torque-controlled knee exoskeleton. In 2017 International Conference on Rehabilitation Robotics (ICORR), pp. 1646–1653, 10.1109/ICORR.2017.8009484. 28814056

[r35] Zhong B, Guo K, Yu H and Zhang M (2022) Toward gait symmetry enhancement via a cable-driven exoskeleton powered by series elastic actuators. IEEE Robotics and Automation Letters 7(2), 786–793. 10.1109/LRA.2021.3130639.

